# On the Estimation of Time Dependent Lift of a European Starling (*Sturnus vulgaris*) during Flapping Flight

**DOI:** 10.1371/journal.pone.0134582

**Published:** 2015-09-22

**Authors:** Oksana Stalnov, Hadar Ben-Gida, Adam J. Kirchhefer, Christopher G. Guglielmo, Gregory A. Kopp, Alexander Liberzon, Roi Gurka

**Affiliations:** 1 Faculty of Engineering and the Environment, University of Southampton, Southampton, Hampshire, SO17 1BJ, United Kingdom; 2 Faculty of Aerospace Engineering, Technion, Israel Institute of Technology, Haifa, 32000, Israel; 3 Boundary Layer Wind Tunnel Laboratory, London, ON N6A3K7, Canada; 4 Department of Biology, Advanced Facility for Avian Research, University of Western Ontario, London, ON, N6A5B7 Canada; 5 School of Mechanical Engineering, Tel Aviv University, Tel Aviv, 69978, Israel; 6 School of Coastal and Marine Systems Science, Coastal Carolina University, Conway, SC 29528, United States of America; Technion - Israel Institute of Technology, ISRAEL

## Abstract

We study the role of unsteady lift in the context of flapping wing bird flight. Both aerodynamicists and biologists have attempted to address this subject, yet it seems that the contribution of unsteady lift still holds many open questions. The current study deals with the estimation of unsteady aerodynamic forces on a freely flying bird through analysis of wingbeat kinematics and near wake flow measurements using time resolved particle image velocimetry. The aerodynamic forces are obtained through two approaches, the unsteady thin airfoil theory and using the momentum equation for viscous flows. The unsteady lift is comprised of circulatory and non-circulatory components. Both approaches are presented over the duration of wingbeat cycles. Using long-time sampling data, several wingbeat cycles have been analyzed in order to cover both the downstroke and upstroke phases. It appears that the unsteady lift varies over the wingbeat cycle emphasizing its contribution to the total lift and its role in power estimations. It is suggested that the circulatory lift component cannot assumed to be negligible and should be considered when estimating lift or power of birds in flapping motion.

## Introduction

Interest in the unsteady aerodynamics of flapping wing flight has been rekindled with the development of micro air vehicles (or MAVs). These MAVs fly at low Reynolds numbers, where many complex flow phenomena take place within the wing boundary layer. For example, separation, transition, and reattachment (of the boundary layer) can occur within a short distance along the surface of the wing and can dramatically affect the performance of the lifting surface. Despite these challenges engineers are not without prior information because nature has produced numerous examples of biological flying machines that have evolved over millions of years to efficiently fly in the low-Reynolds-number regime. One such example is the flapping flight mechanism in which the wings are not only moving forward relative to the air, but also flap up and down, bend, twist and sweep, resulting in a complicated unsteady-aerodynamic motion. Understanding the role of unsteady forces on flapping flight will help in designing more efficient micro-flying vehicles [1].

The flapping motion associated with the unsteady effects generally leads to enhancement of bound vortices on the lifting surface, which eventually detach, convect into the wake, and interact with other vortices [2]. Due to the interaction between the bound vortices on the lifting surface and the vortices in the wake, the performance of an unsteady wing is coupled with the formation and distribution of vorticity shed throughout the wing’s cycle of oscillation [3, 4].

A useful theory to approximate unsteady aerodynamic loads is the unsteady thin airfoil theory. The roots of the theory were originally developed by Glauert [5], who considered simple harmonic motion. However, the complete solution of estimating the time dependent loads for a harmonically oscillating airfoil in inviscid, incompressible flow was first published by Theodorsen [6]. Theodorsen’s work was further complemented by von-Kármán and Sears [7] who simplified the equations and presented the general unsteady thin airfoil theory. In addition to simplifying the equations, von-Kármán and Sears considered also the problem of a thin airfoil moving through a vertical gust field. The effect of unsteady inflow conditions on aerodynamic forces is considered in many applications, for example in helicopter aerodynamics [8, 9].

The role of unsteady forces is significant in estimating the aerodynamic performance of birds flight in flapping motion [10]. As living organisms, birds are subject to selective pressures; as such, one may assume that they operate their wings in a highly efficient manner. This notion is supported by the tendency of birds, as well as many other animals, to operate in a limited Strouhal number range between (0.2 and 0.4 [11, 12]). There are many factors differentiating flapping of a bird’s wings from heaving or pitching of two dimensional airfoils [4]. These differences include the presence of a body, three dimensionality of the wing and its unique motion. Ben-Gida *et al*. [13] compared the formation of steady to unsteady drag in the near wake of a European starling (Sturnus vulgaris), where it was demonstrated that the negative contribution of the unsteady drag component at the transition stages of the wingbeat phase reduces the total drag.

To model the time dependent aerodynamic forces acting on a section of a wing it is natural to start with a quasi-steady approach. The estimation of lift from the PIV measurements behind flying birds is done by applying the classical Kutta-Joukowski theorem, *L* = *ρU*Γ, where *ρ* is the fluid density, *U* is the wind speed, and Γ is the circulation, which is calculated from the vorticity field. Whether the lift is estimated from the Trefftz plane or from the streamwise-normal plane, it is assumed to be quasi-steady [14, 15]. In order to estimate the quasi-steady lift, it is sufficient to capture a portion of, or an entire, wingbeat cycle. Previous work has shown that circulation can be estimated from a single instantaneous vector map [16] or from synchronized velocity maps triggered to match various phases within the wingbeat cycle [17]. Or, using several consecutive velocity maps of which a full wingbeat cycle has been reconstructed, the lift has been estimated using a series of velocity fields capturing the far wake behind a freely flying bird [14, 18]. Yet, analysis incorporating the unsteady effects in lift estimations is lacking. One of the challenges in estimating the evolution of lift over time is the need to measure the wake using a technique that introduces high spatial and temporal resolution over a relatively long period of time. In the present work, the near wake of a freely flying European starling (*Sturnus Vulgaris*) has been selected as a case study to determine unsteady wing aerodynamics [19].

The aim of the present work is to evaluate the unsteady sectional lift of a flapping wing, based on experimental data acquired in the near wake of freely flying European starling (*Sturnus vulgaris*) using long-duration, high-speed Particle Image Velocimetry (hereafter PIV) [20]. In the case of a flapping wing [21], the boundary layer over the wing often experiences an early transition to turbulence due to the unsteady motion and remains attached for higher angles of attack (compared to airfoil in steady condition). Such re-attachment of the boundary layer allows the use of the unsteady thin airfoil theory for lift estimation. As a first approximation the wing is estimated as rigid plate undergoing translational motion using kinematic relations [8] based on von-Kármán and Sears [7] model. Then, using high-speed PIV data, the unsteady portion of the lift is estimated over several wingbeat cycles and compared to the rigid–plate lift approximation.

## Materials and Methods

### Theoretical modelling

The unsteady motion of a viscous fluid about a lifting surface is always accompanied with shedding of vortices into the wake. The addition point of the unsteady thin airfoil theory derivation is through the fact that the circulation around the surface is varying continuously and two-dimensional vorticity is shed off into the wake, thus allowing the use of planar wake assumption and ignoring the effect of wake deformation. In the far wake, the vortices roll-up under their own self-induced velocities to form complex wake patterns. Despite this difficulty, the contribution of the wake vortices is not significant since the influence of the shed vortices reduces with increasing distance. However, in the near wake, where the PIV measurements were conducted, the flow structures are simplified, thus allowing the use of planar wake assumption, ignoring the effects of wake deformation. Here we derive the unsteady thin airfoil theory. For clarity, we use the unsteady Bernoulli equation to evaluate the time-dependent loads. This derivation, to the best of our knowledge, has not been reported elsewhere.

**Fig 1:**
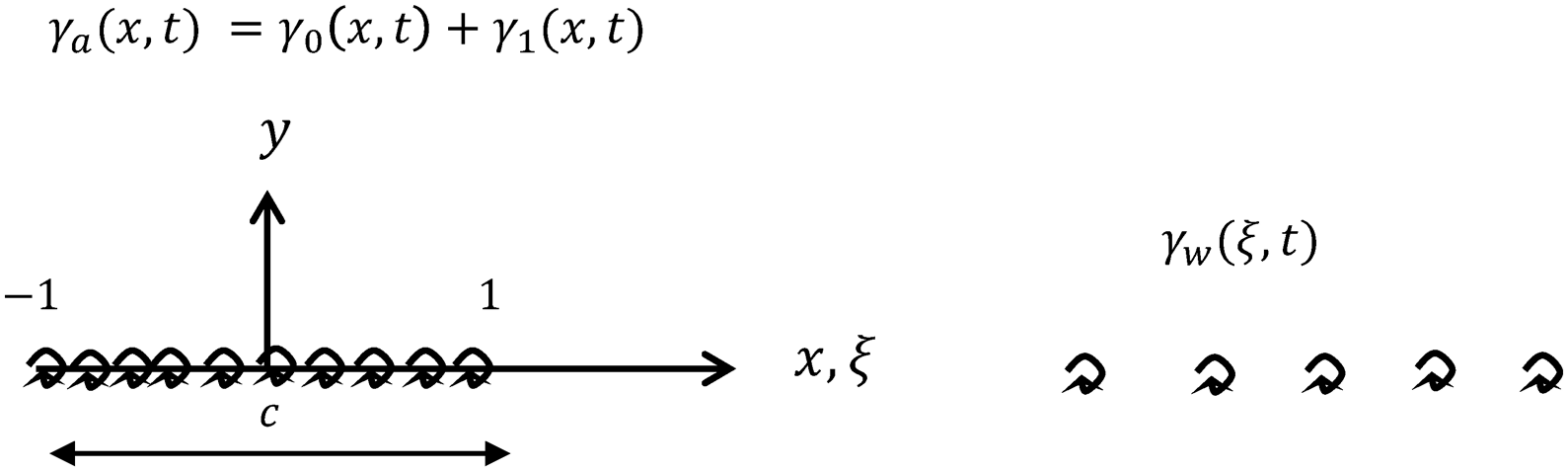
Auxiliary diagram showing notation employed.

We follow the classical definition of the unsteady thin airfoil theory [6, 7] notation where the chord is equal to *c* = 2 (see Fig 1). The leading-edge of the lifting surface is placed at *x* = −1 and trailing-edge at *x* = 1, whereas the mid chord is placed at the origin of the coordinate system at *x* = 0. The *y*-axis is perpendicular to the flow and the *z*-axis is in the spanwise direction of the wing.

The analysis is initiated by assuming that the vorticity distribution bound to the wing section *γ*_*a*_(*x*, *t*) is the sum of the quasi-steady vorticity distribution *γ*_0_(*x*, *t*) that would have been produced in quasi-steady motion and a wake-induced vorticity distribution *γ*_1_(*x*, *t*)

$$\gamma_{a}\left(x,t\right)=\gamma_{0}\left(x,t\right)+\gamma_{1}\left(x,t\right).$$ (1)

The total circulation about the airfoil due to both the quasi-steady vorticity distribution and that induced by the wake is

$$\Gamma_{a}=\Gamma_{0}+\Gamma_{1}$$ (2)

where

$$\Gamma_{0}=\int_{-1}^{1}\gamma_{0}\left(x,t\right)\;dx$$ (3)

and

$$\Gamma_{1}=\int_{-1}^{1}\gamma_{1}\left(x,t\right)\;dx.$$ (4)

One of the fundamental assumptions in inviscid aerodynamics is that, according to the Kelvin circulation theorem, the total circulation of a system is equal to zero. The total circulation about the wing section is a sum of the quasi-steady circulation Γ_0_, that would be produced if the total circulation would not have been affected by the presence of the wake, and the wake-induced circulation Γ_1_. When the circulation around the wing section Γ_*a*_ is balanced with the circulation produced by the wake Γ_*w*_ at every time step *t*

$$\frac{\partial }{\partial t}\int_{-1}^{1}\gamma_{0}\left(x,t\right)\;dx+\frac{\partial }{\partial t}\int_{-1}^{1}\gamma_{1}\left(x,t\right)\;dx+\frac{\partial }{\partial t}\int_{1}^{\infty }\gamma_{w}\left(\xi ,t\right)\;d\xi =0.$$ (5)

#### Wake-induced circulation

The effect of the wake vortices is evaluated in accordance with the thin airfoil theory, where the Joukowski’s conformal transformation is used to transform a circle in the *z*′ plane to an airfoil at the *z* plane. The transformation relating the two planes is

$$z=\frac{1}{2}\;(z^{{}^{\prime }}+\frac{1}{z^{{}^{\prime }}}).$$ (6)

A single vortex element defined as Γ′ at a distance of *ξ* in the *z*-plane is located in the *z*′-plane at a distance of *η*. To create a unit circle which transforms into a flat plate in the *z* plane, another vortex element with an opposite sign has to be introduced inside the unit circle at a symmetric point, which is 1/*η*. Thus, along a unit circle the induced velocity magnitude is

$$v_{\theta }=\frac{\Gamma^{{}^{\prime }}}{2\pi |z^{\prime }-\eta |}-\frac{\Gamma^{{}^{\prime }}}{2\pi |z^{\prime }-1/\eta |}$$ (7)

which is equal to

$$v_{\theta }=\frac{\Gamma^{{}^{\prime }}}{2\pi }|\frac{\eta -\frac{1}{\eta }}{z^{\prime 2}-z^{\prime }\;\left(\eta +\frac{1}{\eta }\right)+1}|.$$ (8)

Since *z*′ is placed on the unity circle, the trigonometric identity that describes the unit circle is *z*′ = cos *θ* + *i* sin *θ*, resulting in

$$v_{\theta }=\frac{\Gamma^{\prime }}{2\pi }\;(\frac{\sqrt{\xi^{2}-1}}{\xi -cos\theta }).$$ (9)

At the trailing-edge cos*θ* = 1, thus the induced velocity at the trailing-edge is

$$v_{\theta_{TE}}=\frac{\Gamma^{\prime }}{2\pi }\sqrt{\frac{\xi +1}{\xi -1}}.$$ (10)

According to the Kutta condition, the total circulation around an airfoil is such that, at any instance, the flow velocity is tangential to the trailing-edge. In absence of additional information with respect to the bound circulation, as was implemented in the work of Ford and Babinsky [22], this is well established assumption. Therefore, to meet the this condition the tangential velocity at the trailing-edge is subtracted from Eq (9), the total tangential velocity on the airfoil is

$$v_{\theta }=\frac{\Gamma^{\prime }}{2\pi }(\frac{\sqrt{\xi^{2}-1}}{\xi -cos\theta }-\sqrt{\frac{\xi +1}{\xi -1}})=-\frac{\Gamma^{\prime }}{2\pi }(\frac{1-cos\theta }{\xi -cos\theta })\;\sqrt{\frac{\xi +1}{\xi -1}}.$$ (11)

The relationship between the velocity *v*_*θ*_ and the vorticity distribution over the airfoil *γ*(*x*) according to thin airfoil theory is given by the formula *γ*(*x*) = −2*v*_*θ*_/ sin *θ*. Further, using the trigonometric relationship *x* = cos *θ* and $\sqrt{1-x^{2}}=\sin\theta$, the effect of induced vorticity from a single vortex at a point *ξ* in the wake can be written as

$$\gamma_{1}^{\prime }\left(x,t\right)=\frac{\Gamma^{\prime }}{\pi (\xi -x)}\sqrt{\frac{\xi +1}{\xi -1}}\sqrt{\frac{1-x}{1+x}}$$ (12)

where ′ denotes a single vorticity element. The effect of the single element of vorticity Γ′ can be replaced by *γ*_*w*_(*ξ*, *t*)d*ξ*. From Eq (12) we can derive an expression for the induced vorticity of the entire wake

$$\gamma_{1}\left(x,t\right)=\frac{1}{\pi }\sqrt{\frac{1-x}{1+x}}\int_{1}^{\infty }\frac{\gamma_{w}\left(\xi ,t\right)}{\xi -x}\sqrt{\frac{\xi +1}{\xi -1}}\;d\xi$$ (13)

resulting in wake-induced circulation

$$\Gamma_{1}=\int_{1}^{\infty }\gamma_{w}\left(\xi ,t\right)\;(\sqrt{\frac{\xi +1}{\xi -1}}-1)\;d\xi .$$ (14)

#### Unsteady thin airfoil theory

Kármán and Sears [7] applied the principle that, in accordance with the Newton’s second law, the product of density and the rate of change of the total momentum at any instance is equal to the lift. In the current study, an estimation of the lift due to an unsteady motion is based on the integration of normal pressure difference along the chord

$$L\left(x,t\right)=\int_{-1}^{1}\Delta p(x,t)\;dx.$$ (15)

The pressure difference Δ*p*(*x*, *t*) in terms of the chordwise vorticity distribution *γ*_*a*_(*x*, *t*) is expressed by the unsteady Bernoulli equation [23]

$$\Delta p\left(x,t\right)=\rho U\gamma_{a}\left(x,t\right)+\rho \frac{\partial }{\partial t}\int_{-1}^{x}\gamma_{a}\left(x_{0},t\right)\;dx_{0}.$$ (16)

Thus, the lift can be written as

$$L\left(x,t\right)=\rho U\int_{-1}^{1}\gamma_{a}\left(x,t\right)\;dx+\rho \int_{-1}^{1}\frac{\partial }{\partial t}\int_{-1}^{x}\gamma_{a}\left(x_{0},t\right)\;dx_{0}\;dx.$$ (17)

Integration by parts of the second integral on the right hand side of Eq (17) yields

$$\begin{array}{cc} \rho \int_{-1}^{1}\frac{\partial }{\partial t}\int_{-1}^{x}\gamma_{a}\left(x_{0},t\right) & \;dx_{0}\;dx=\\ & \rho \frac{\partial }{\partial t}([x\int_{-1}^{x}\gamma_{a}\left(x_{0},t\right)\;dx_{0}]\;{|}_{-1}^{1}-\int_{-1}^{1}\gamma_{a}\left(x,t\right)x\;dx) \end{array}$$ (18)

where it is recognized that

$$[x\int_{-1}^{x}\gamma_{a}\left(x_{0},t\right)\;dx_{0}\;]{|}_{-1}^{1}=\int_{-1}^{1}\gamma_{a}\left(x,t\right)\;dx.$$ (19)

The lift terms in Eq (17) can then be rearranged as

$$\begin{array}{cc} L= & \rho U\int_{-1}^{1}\gamma_{0}\left(x,t\right)dx-\rho \frac{\partial }{\partial t}\int_{-1}^{1}x\gamma_{0}\left(x,t\right)\;dx+\rho \frac{\partial }{\partial t}\int_{-1}^{1}\gamma_{0}\left(x,t\right)\;dx\\ & +\rho U\int_{-1}^{1}\gamma_{1}\left(x,t\right)\;dx-\rho \frac{\partial }{\partial t}\int_{-1}^{1}x\gamma_{1}\left(x,t\right)\;dx+\rho \frac{\partial }{\partial t}\int_{-1}^{1}\gamma_{1}\left(x,t\right)\;dx. \end{array}$$ (20)

The first term in Eq (20)

$$L_{0}=\rho U\int_{-1}^{1}\gamma_{0}\left(x,t\right)\;dx$$ (21)

is the quasi-steady Kutta-Joukowski lift component. In permanently maintained flow conditions this would be the only lift component. In unsteady flow conditions the quasi-steady lift component only partially contributes to the total lift and it is determined by evaluating instantaneous angle of attack. The second term in Eq (20)

$$L_{1}=-\rho \frac{\partial }{\partial t}\int_{-1}^{1}x\gamma_{0}\left(x,t\right)\;dx$$ (22)

is the apparent (or added mass) lift component that accounts for the reaction due to the mass of fluid directly accelerated by the wing. Following the von-Kármán and Sears discussion on time derivative, the fifth term of Eq (20) is equal to

$$\begin{array}{cc} \rho \frac{\partial }{\partial t} & \int_{-1}^{1}x\gamma_{1}\left(x,t\right)\;dx=\rho \frac{\partial }{\partial t}\int_{1}^{\infty }\gamma_{w}\left(\xi ,t\right)\;d\xi +\rho \frac{\partial }{\partial t}\int_{1}^{\infty }(\sqrt{\xi^{2}-1}-\xi )\gamma_{w}\left(\xi ,t\right)\;d\xi \\ & =\rho \frac{\partial }{\partial t}\int_{1}^{\infty }\gamma_{w}\left(\xi ,t\right)\;d\xi +\rho U\int_{1}^{\infty }\;(\sqrt{\frac{\xi +1}{\xi -1}}-1-\frac{1}{\sqrt{\xi^{2}-1}})\gamma_{w}\left(\xi ,t\right)\;d\xi \\ & =\rho \frac{\partial }{\partial t}\int_{1}^{\infty }\gamma_{w}\left(\xi ,t\right)\;d\xi +\rho U\int_{-1}^{1}\gamma_{1}\left(\xi ,t\right)\;d\xi -\rho U\int_{1}^{\infty }\frac{\gamma_{w}\left(\xi ,t\right)}{\sqrt{\xi^{2}-1}}\;d\xi . \end{array}$$ (23)

It should be noted, that the third term of Eq (23) is the wake-induced lift

$$L_{2}=\rho U\int_{1}^{\infty }\frac{\gamma_{w}\left(\xi ,t\right)}{\sqrt{\xi^{2}-1}}\;d\xi .$$ (24)

Evaluation of *L*_2_ term requires either an assumption about the unsteady motion, keeping track of the shed vorticity into the wake, or including the shed vorticity through a convolution integral. The fourth term in Eq (20) is cancelled with the second term of Eq (23). Adding the third and sixth terms in Eq (20) with the first term in Eq (23) results in the Kelvin theorem (see Eq (5)), therefore, this summation is zero. These leads to the time dependent lift (Eq (20)) that can be written as the sum of three terms

$$L=\rho U\Gamma_{0}-\rho \frac{\partial }{\partial t}\int_{-1}^{1}x\gamma_{0}\left(x,t\right)\;dx+\rho U\int_{1}^{\infty }\frac{\gamma_{w}\left(\xi ,t\right)}{\sqrt{\xi^{2}-1}}\;d\xi .$$ (25)

Eq (25) is the result of von-Kármán and Sears [7]. The first term is the quasi-steady lift *L*_0_ produced by the bound vorticity. The second term *L*_1_ represents the apparent (added) mass contribution to lift component and it results from the inertia of the fluid moving with the lifting surface. The third term *L*_2_ is the induced lift component that produced by the wake vorticity. It should be noted that the contribution to the lift due to wake-induced vorticity *γ*_1_(*x*, *t*) is cancelled out in the derivation of the equations.

This result coincides with Theodorsen [6], who suggested to divide the time dependent lift into circulatory and non-circulatory components, namely *L*_*C*_ and *L*_*NC*_, respectively. The non-circulatory lift can be referred as the virtual mass effect or the acceleration reaction term [24] and the circulatory lift component is generated from the vortical flow around the lifting surface. We should point out that the non-circulatory lift term, *L*_*NC*_, which is related to the added mass lift, is identical to the *L*_1_ term presented by von-Kármán and Sears [7] (see Eq (22)), i.e. *L*_*NC*_ = *L*_1_. The circulatory lift term *L*_*C*_ is equal to the sum of the two remaining lift components, these are the quasi-steady lift and the wake-induced lift, i.e., *L*_*C*_ = *L*_0_+*L*_2_.

### Experimental Apparatus

#### Wind tunnel

The experiments reported herein were conducted in a hypobaric climatic wind tunnel at the Advanced Facility for Avian Research (AFAR) at the University of Western Ontario. A detailed description of the wind tunnel, the experimental technique and the bird is given by Ben-Gida *et al*. [13] and Kirchhefer *et al*. [19]. Herein, we provide a short description, for brevity. The wind tunnel is closed–loop type with an octagonal test section. The cross-sectional area is 1.2 m^2^, preceded by a 2.5:1 contraction ratio. The width, height and length of the test section are 1.5 m, 1 m, and 2 m, respectively. The control of speed, pressure, temperature, and humidity in the wind tunnel enables simulation of flight conditions at high altitudes as experienced by birds during long distance migratory conditions. The bird is introduced into the test section through a partition that is located between the downstream end of the test section and the diffuser. The turbulence intensity measured by the hot-wires was lower than 0.3% over the entire test section with a uniformity of 0.5%. A fine size net was placed at the upstream end of the test section to prevent the bird from entering the contraction area. The flight conditions reported in this work correspond to atmospheric static pressure, a temperature of 15°C, and relative humidity of 80%.

#### The Bird—European Starling

**Fig 2:**
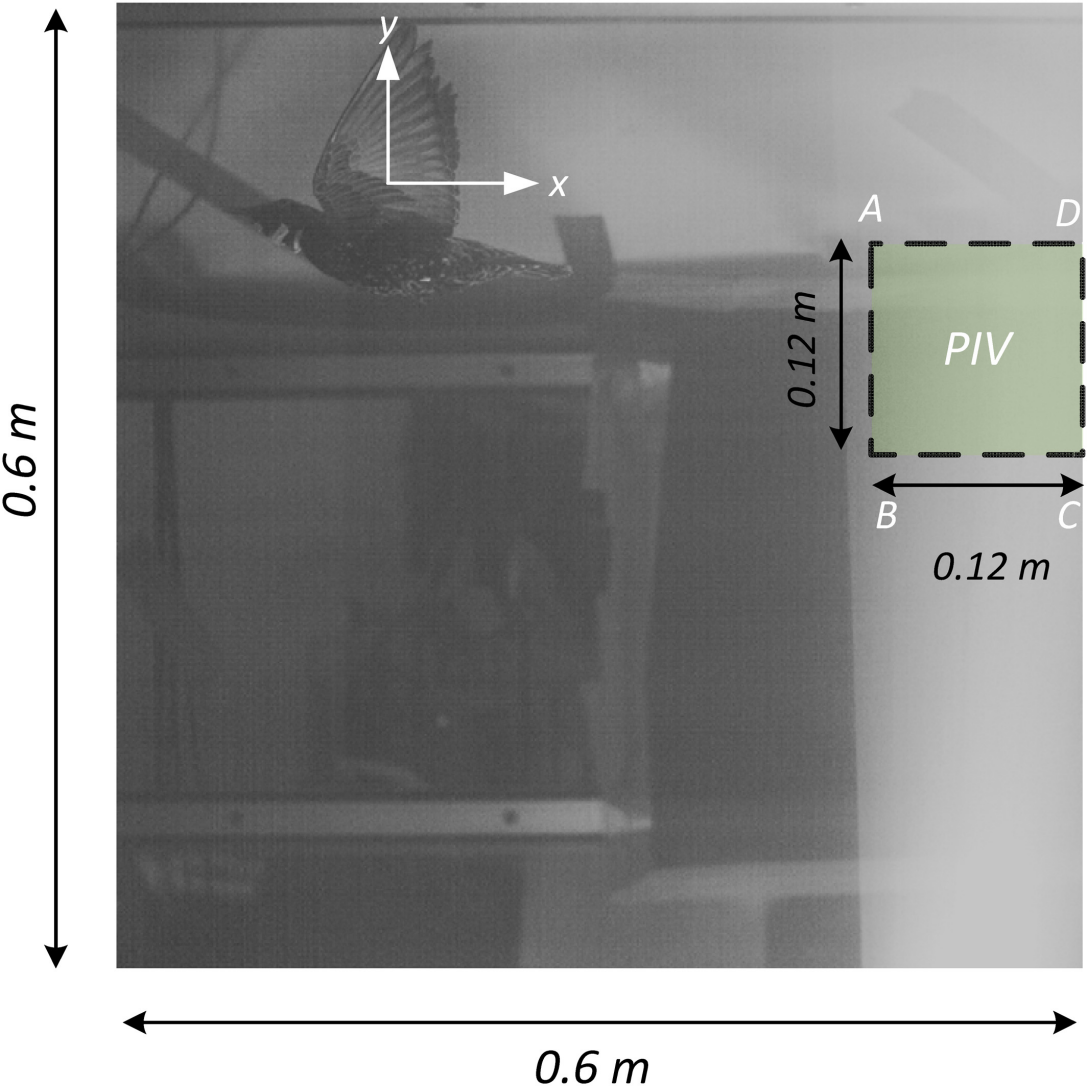
The large image shows the kinematic camera field of view and the small window marked ‘PIV’ is the PIV camera field of view.

The measurements (as illustrated in Fig 2) were sampled in the wake of a freely flying European starling (*Sturnus vulgaris*) that was trained to fly in the AFAR wind tunnel. The bird’s wings had an average chord of *c* = 6 cm, a maximum wingspan of *b* = 38.2 cm (*b*_*semi*_ = 19.1 cm) and an aspect ratio (wingspan squared divided by the wings lifting area) of 6.4. The wind speed was set to *U*_∞_ = 12 m/s. The wingbeat frequency, *f*, was 13.3 Hz on average, and the average peak-to-peak wingtip vertical amplitude, *A*, was 28 cm. These quantities correspond to a chord-based Reynolds number of 4.8 × 10^4^, a Strouhal number, *St* = *Af*/*U*_∞_ = 0.3, and a reduced frequency, *k* = *πfc*/*U*_∞_ = 0.2. The bird’s mass was 78 g with a lateral body width of 4 cm. Specially designed safety goggles (Yamamoto Cogaku Co. model YL600) were adjusted to the bird while flying in the tunnel. In addition, a collection of optoisolators operated by six infrared transceivers were integrated into the PIV system in order to prevent direct contact between the bird and the laser sheet. The optoisolators triggered the laser only when the bird was flying upstream the PIV field of view. All animal care and procedures were approved by the University of Western Ontario Animal Use Sub-Committee (protocols 2006-011, 2010-216).

#### Long duration time resolved PIV

Flow measurements were taken using the long-duration time-resolved PIV system developed by Taylor *et al*. [12]. The PIV system consists of a 80 W double-head diode-pumped Q-switched Nd:YLF laser at a wavelength of 527 nm and two CMOS cameras (Photron FASTCAM-1024PCI) with spatial resolution of 1024 × 1024 pixel^2^ at a sampling rate of 1000 Hz. The PIV system is capable of acquiring image pairs at 500 Hz using two cameras for a continuous period of 20 minutes. Olive oil aerosol particles, 1 *μ*m in size on average [25] were introduced into the wind tunnel using a Laskin nozzle from the downstream end of the test section so that it did not cause a disturbance to the flow in the test section or to the bird. The system is designed to work either in a 2D or Stereo mode. Herein, we used one camera for the PIV whilst the other camera was used for measuring the wingbeat kinematics simultaneously with the PIV. The PIV camera’s field of view was 12 × 12 cm^2^ corresponding to 2*c* × 2*c*. The velocity fields were computed using OpenPIV [20] using 32 × 32 pixel^2^ interrogation windows with 50% overlap, yielding a spatial resolution of 32 vectors per average chord, equal to 1.8 vectors per millimetre. In the current experiments, 4,600 vector maps were recorded, and out of this dataset 650 vector maps contained features of the near wake behind the starling’s wing. The measured wake was located 4 wing chord lengths behind the right wing. The wake was sampled in the streamwise-normal plane at 2 ms intervals (500 Hz), therefore, both the downstroke and the upstroke phases were temporally resolved.

#### Kinematic measurements

**Fig 3:**
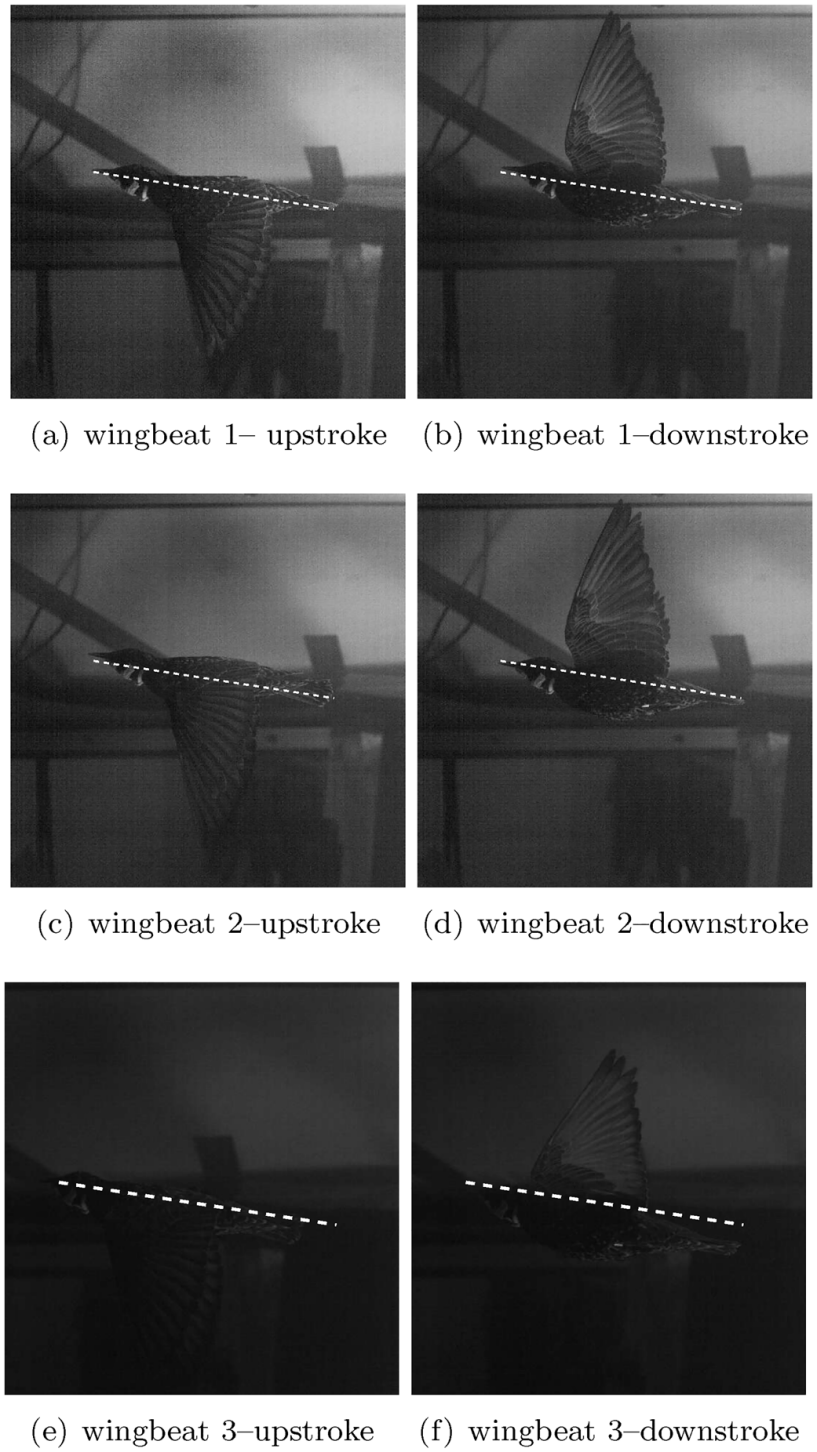
Side view of the European starling at the wind tunnel. The white line is placed to provide spatial reference of the bird’s body. The white line is inclined at 8.8° with the free-stream velocity. The left and right images correspond to beginning of the downstroke and upstroke, respectively.

To relate the wake measurements to the kinematic motion of the bird’s wings, an analysis of the kinematic motion has been undertaken. The simultaneous measurements also enable a point of comparison between the estimation of lift through the unsteady thin airfoil theory and lift calculation using the momentum equation for viscous flows. The field of view by the kinematic CMOS camera is 9*c* × 9*c* (corresponding to 54 × 54 cm^2^). Fig 2 depicts a sample image of the Starling flying in the wind-tunnel as captured by the camera. The box marked with the ‘PIV’ label indicates the location of the measured velocity fields from the PIV system. During the time of data acquisition, the bird maintained its position with respect to the PIV field of view. Fig 3 depicts different positions of the starling during the three wingbeats, where it is shown that the starling maintained its vertical position along this wingbeat cycle.

In addition, a floor-mounted camera operating at 60Hz was used to record the spanwise position of the bird with respect to the laser sheet illumination. These images provided the identification of the measured PIV plane and its location with respect to the wing such that the wake velocity field associated with the spanwise location across the wing was 0.15*b*/2 from the root. The floor-mounted camera was not synchronized with the PIV or the kinematic measurements, so the two time histories were synchronized manually based on the presence of the laser light-sheet in the images. Once synchronized, spanwise positions were assigned to the wake data acquired at 500 Hz based on interpolation from the simultaneously recorded spanwise positions.

The kinematic parameters which where estimated for this study were the angle of attack and the flapping angle. The wing root and wing tip were used as points of reference for the position and velocity of the bird. The motion of the wing was expressed by its angular position, *θ*, in a cylindrical coordinate system. Their positions have been calculated using calibrated images since the spanwise location was pre-determined from calibrating the light sheet location. The procedure assumed that the wings were fully extended at all times. As a result, with a known wing root and tip coordinates and known wing length, we were able to calculate various kinematic features [19]. The flapping angle was obtained by estimating the wing oscillation and measuring the peak-to-peak amplitude, which was on average equivalent to 74 degrees. This resulted in flapping angle amplitude of 37 degrees at the wing tip.

#### Error estimation

An error analysis based on the root-sum-of-squares method has been applied to the velocity data and the wing kinematics. The resultant error of the velocity field that was obtained from the PIV images considered the error contributions due to image magnification, lens distortion, the time between two consecutive laser pulses and the accuracy of the wind speed in the wind–tunnel. The vorticity was estimated using the high order least-squares-interpolation square finite difference technique [26] and for the lift we have used a first order numerical integration scheme, which, for the current case, a cumulative summation function (realized with a standard library function cumsum) was used. The errors were estimated as: 2.5% for the instantaneous velocity values, 12% for the instantaneous vorticity and 3–5% for the lift values [27]. The error introduced in the kinematic analysis resulted from the spatial resolution of the image and the lens distortion leading to an estimated error of 5% in the wing displacements.

## Results and Discussions

The PIV flow field measurements and the bird’s kinematics were each analysed separately in order to estimate the time dependent component of the lift generated by the flapping motion of the starling using two approaches. Linear lift theory (see the theoretical modelling section) was used to estimate the lift from the bird’s kinematics, whereas a viscous flow theory, derived by Wu [28] and applied by Panda and Zaman [29], was implemented to estimate the lift from the near wake flow fields measured by the PIV. The present work considers a comparison between the linear theories [6, 7] and the viscous flow theory [28], for the lift generated by the starling. Both approaches will emphasize the role of the time dependent lift components.

The bird kinematics and the near wake velocity field were captured simultaneously while the starling was flying in the tunnel and flapping its wings continuously. During flapping, birds generate lift and thrust. The lift is necessary to support the bird’s body weight and the thrust is required to overcome the drag. The data presented herein correspond to no-maneuver and no-acceleration conditions. The spatial location of the bird’s body at the beginning of the downstroke and upstroke phases of flight are shown in Fig 3 for three consecutive wingbeats. It can be seen that the bird does not accelerate in the streamwise or vertical directions [13]. Hence, the following kinematic analysis can be performed assuming negligible acceleration.

### Estimation of time-dependent lift from the bird kinematics

As presented in the theoretical modelling section, the linear theories were derived within the framework of potential theory, which assumes inviscid fluid with small disturbances and a plane wake. One can use such theories to estimate the time dependent lift components from the kinematics of a wing section with relatively good precision [8]. We choose to use the guidelines provided by Leishman [8] and estimate the time dependent lift from the bird’s kinematics.

**Fig 4:**
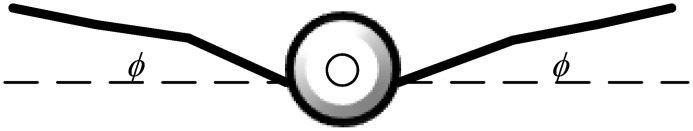
Auxiliary diagram of the flapping motion with the definition of the flapping angle. View in the direction of the flow.

Using the aforementioned unsteady thin airfoil theory, we estimate the lift generated by the flapping wings motion of the European starling, as captured through the wings kinematic images. For simplicity, we describe the kinematics of a flapping wing by a purely two-dimensional plunging motion, which involves a heaving up and down of the wing section that results in a variation of the effective angle of attack [8]. In such motion the variation of the vertical displacement with time can be described as

$$h_{f}\left(t\right)=h_{f_{0}}\cos\left(\omega t\right)$$ (26)

where *h*_*f*_0__ is the plunging motion amplitude and *ω* is the angular velocity. For a flapping wing the plunging amplitude *h*_*f*_0__ is a function of the distance from the shoulder joint. Assuming the elastic deformation of the wing is negligible we can describe the plunging amplitude as linearly increasing function towards the wing tip. Thus, the vertical displacement amplitude at spanwise distance *η* = 0.15*b*/2 from the wing root can be reduced to *h*_*f*_0__ = *η*cos(*ϕ*), where *ϕ* is the wing tip angle, as depicted in Fig 4. Kinematic images of the starling [19] depicted the wings as they oscillate in a periodic manner, where the range of the angular positions is −55° < *ϕ* < 19°.

Due to the unique bone and muscle structure of the bird’s wing, Dhawan [28] suggested that during flapping flight the inner part of the wing experiences less twisting motion than the outer part which accounts for most of the thrust production. Therefore, we assume that the variation of the local angles of attack at the inner part of the wing are small compared to those at the outer part. Following that, we suggest describing the effective angle of attack as a result of the horizontal free-stream velocity and the vertical velocity component due to plunging motion, $\alpha_{e}=\tan^{-1}\left(\dot{h}\left(t\right)/U_{\infty }\right)$. By assuming small angles of attack we can simplify the effective angle of attack to $\alpha_{e}\approx \left(\dot{h}\left(t\right)/U_{\infty }\right)$. Consequently, the quasi-steady lift component can be estimated by the thin airfoil theory [30], where the non-dimensional lift coefficient is a function of the effective angle of attack and the corresponding lift component is equal to

$$L_{0}=\pi \rho U^{2}c\;[\frac{\dot{h}\left(t\right)}{U}].$$ (27)

Following the unsteady thin airfoil theory [6, 7], the added-mass lift component is a result of flow acceleration, and thus arises from the unsteady term in the Bernoulli equation that accounts for the pressure force required to accelerate the fluid in the the vicinity of the wing. For the wing section moving normal to its surface, the non-circulatory fluid force acting on the surface is equal to the product of apparent mass and acceleration. Thus, a body moving in an unsteady motion must overcome acceleration in addition to its own inertial force. Therefore, the apparent mass (or non-circulatory [6]) lift component can be estimated from the kinematic motion as

$$L_{1}=\pi \rho U^{2}\frac{c^{2}}{4}\;[\frac{\ddot{h}\left(t\right)}{U^{2}}]$$ (28)

where $\dot{h}\left(t\right)$ is the time derivative of vertical displacement [8].

Here, the effect of the wake-induced lift component *L*_2_ is determined by assuming harmonic motion [6] at a frequency *ω*, yielding *γ*_*w*_(*ξ*, *t*) = *ge* ^*iω*(*t* − *ξ*/*U*)^. Using Theodorsen’s function *C*(*k*), which accounts for the effect of the shed vortices on the unsteady aerodynamic loads, we can calculate the wake-induced lift component as

$$L_{2}\left(t\right)=\left(C\left(k\right)-1\right)L_{0}$$ (29)

where the definition of Theodorsen’s function is [6]

$$C\left(k\right)=\frac{H_{1}^{\left(2\right)}\left(k\right)}{H_{1}^{\left(2\right)}\left(k\right)+iH_{0}^{\left(2\right)}\left(k\right)}.$$ (30)

Here $H_{n}^{\left(2\right)}=J_{\nu }-iY_{\nu }$ is the Hankel function of the reduced frequency *k*, where *J*_*ν*_ and *Y*_*ν*_ are Bessel functions of the first and second kind, respectively. The lift reduction function *C*(*k*) falls gradually to value of 0.5 as *k* goes to infinity. The effect of *C*(*k*) as producer of phase lag takes over very quickly, where the maximum rotation of the vector occurs around *k* = 0.2. It should be noted that the reduced frequency values used by many small passerines, such as the European starling, is in the range of 0.1 < *k* < 0.3 [31]. Apparently, these small passerines fly in the region where the effect of the lift reduction function is the strongest.

**Fig 5:**
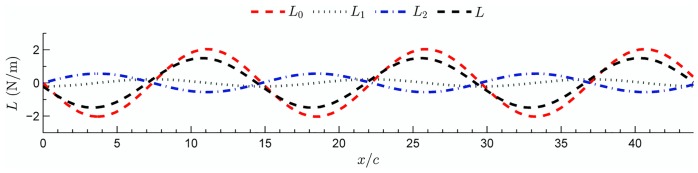
Time dependant components of unsteady lift terms, as estimated from the wing kinematics. The terms *L*_0_, *L*_1_, *L*_2_, are estimated from Eqs 27, 28 and 29, respectively. The term *L* = *L*_0_+*L*_1_+*L*_2_ is the total lift.

Fig 5 shows the time variation of the three lift components (*L*_0_, *L*_1_ and *L*_2_). The sum of the three components correspond to the total time dependent lift, *L*, generated during the flapping motion. It appears that the non-circulatory (or added mass) contribution to lift is the smallest among the three components. Yet, this contribution is not negligible and, in fact, is equal to about half of the the lift generated by the induced-wake. Both components are significantly smaller compared to the quasi-steady lift component. According to the harmonic assumption the wake-induced lift is in anti-phase to the quasi-steady lift component. The generation of the circulatory lift components comprises of two terms that in fact are counter to each other. The theory explains the negative work done by the induced vorticity during the upstroke phase as the wing approaches maximum value of the lift. Rival *et al*. [32] utilized similar principles to study the effect of leading-edge vortex on the formation of dynamic stall over an airfoil.

### Estimation of the circulatory time-dependent lift component from the bird’s near wake vorticity field

The unsteady thin airfoil theory is a useful tool that provides a good approximation of the time dependent aerodynamic loads. Nevertheless, the theory, which bounds to two-dimensional inviscid flows, underestimates loads in complex flows where viscous effects cannot be ignored. One of the methods for estimating aerodynamic loads is based on the flow field at the wake region. PIV provides high resolution spatial data with sufficient accuracy that enables the estimation of such loads from the wake of bluff bodies [27]. However, despite many advances in the current state-of-the-art in experimental diagnostics, practical application of PIV to estimate time dependent forces from wake flow-field measurements are challenging. These efforts are limited to 2D planes. This limitation is mainly due to the fact that the 3D flow-field measurements are restricted by relatively small volume size and low Reynolds number flows. Thus, the most practical approach to estimate the time dependent lift component is from 2D plane measurements.

Within the avian research community, the most common 2D approach is concerned with measurements of the vorticity field in the far wake Trefftz plane and application of the Kutta-Joukowski quasi-steady theorem in order to estimate the lift. This approach is based on the classical assumption that the vortex lines behind a lifting surface roll-up when they propagate downstream into the wake, and they bundle into tip vortices. Thus, the far wake is dominated by the tip vortices. This approach is appealing as the wake structures can be captured by a single plane, provided these measurements are acquired in the far wake. However, measurements conducted in the Trefftz plane are highly inaccurate due to plane normal velocity component and may lead to significant errors that are hard to ignore [33]. Furthermore, these measurements allow estimation of only the mean quasi-steady total lift and not the time dependent evolution of the lift.

The second 2D approach is concerned with measurements of the flow field in the streamwise plane in the near or far wake. A brief summery of the PIV measurement acquired in the wake of freely flying birds can be found in Fig 1 of Kirchhefer *et al*. [19], amongst which are flow measurements in the wake of Thrush Nightingale [14], Robin [18], Swift [16] and bats [34, 35]. PIV measurements in the streamwise plane are considered to be accurate, with some errors related to the spanwise velocity component [27]. As has been indicated previously, one of the first applications of PIV technique to estimate the lift from the wake of freely flying bird is attributed to Spedding *et al*. [14]. In this work, the lift was estimated based on quasi-steady Kutta-Joukowski thin airfoil theory. This simplified approach, which has been followed by many other researchers, neglects the effects of added mass and wake-induced vorticity on the time–dependent lift components.

In the current work, the near wake flow-fields were captured simultaneously with the bird’s kinematic motion, shown in Fig 3 and discussed in the previous section, allowing one to relate the wake flow-field structures to the bird’s kinematic motion. The PIV measurements were taken at the inner part of the right wing (from the bird’s perspective), at a spanwise distance of *z* = 0.15*b*/2 from the wing root. In order to shed light on the wake structures that manifest the lift, a visualization of the entire wingbeat during a single flapping cycle is performed by generating a wake composite image from multiple PIV realizations. A similar approach was first applied by Spedding *et al*. [14] in which PIV measurements (from separate wingbeat cycles) were arranged to represent a complete and representative wavelengths of the wake.

The wake composite is formed by plotting sequential PIV realizations where each image is offset to one-another in the streamwise direction. The offset of the PIV images is calculated as *U*_∞_Δ*t* ⋅ *n*. The generation of a wake composite provides a useful tool for observing the time-series of measurements representing the wake of a wingbeat cycle. The procedure was performed using the PIV flow-fields collected at a sampling rate of 500 Hz, which is significantly higher than the bird’s flapping frequency of 13 Hz. Therefore, a pattern of vorticity appearing in one frame also appears in the consecutive frame—only phase-shifted. The wake structures that appear ‘downstream’ in the wake composite image happen earlier in time, while the structures that appear ‘upstream’ in the composite actually happen later in time. In a sense, the generation of the wake composite image invokes Taylor hypothesis [36] in which the characteristics of the flow are advected through the field of view, where the offset of one image to the next is based on the free stream speed. It should be noted that the typical offset of *U*_∞_Δ*t* ⋅ *n* between images is ∼ 0.4*c* and an instantaneous PIV measurement has a spatial dimension of 2*c*. Therefore, at any location in the wake composite image, there are several overlapping images that can be used to ascertain the instantaneous wake characteristics over the streamwise distance of 2*c* to compare with the wake composite at the same location.

The wake features are shown through fluctuating velocity and vorticity fields, where the spanwise vorticity is defined as follows

$$\omega_{z}\left(t\right)=\frac{\partial v}{\partial x}-\frac{\partial u}{\partial y}$$ (31)

and is evaluated directly from the PIV flow fields using a least squares differentiation scheme. Here *u* and *v* are streamwise and transverse velocity components, respectively.

As it was indicated by Poelma *et al*. [37], there are several approaches to evaluate the aerodynamic force on an immersed body from the velocity field. In this study, estimation of time dependent lift generated during the flapping motion of the starling is evaluated from the near wake velocity maps by utilizing the viscous flow approach derived by Wu [28], which is based on the Navier-Stokes equations [24, 38]. Wu’s generalized formulation, which conveniently describes the aerodynamic forces exerted by a fluid on a solid body immersed in, and moving relative to the fluid, is equal to inertial force due to the mass displaced by the solid body and a term proportional to the time of change of the first moment of the vorticity field [28, 39, 40]. This yields

$$L\left(t\right)=-\rho \frac{d}{dt}\;[\int \int \;x\omega_{z}\left(t\right)\;dxdy]+m^{\prime }\frac{dU}{dt}$$ (32)

where *m*′ is the mass of the fluid displaced by the solid body and the term ∬*xω*_*z*_ *dxdy* is the first *x*-moment of the vorticity. One can immediately recognize that the second term in Eq (32) is inertia force of the fluidic body [37, 40, 41], which correspond to *L*_1_ in the von-Kármán and Sears notation. The equation derived by Wu [28] is based on the principle that, if the vorticity distribution over the entire flow field were known, the force could be evaluated accurately. However, it should be noted that with the current tools it was not possible to estimate the non-circulatory (or *L*_1_) contribution to lift from the wake flow-field. Thus, similarly to the approach made by Panda and Zaman [29], the second term in Eq (32) was neglected.

Although the first lift term in Eq (32) may, for utility and convenience, be divided further into *L*_0_ and *L*_2_ components, such division is to some extent arbitrary. According to this approach, the circulatory lift *L*_*c*_(*t*) is equal to the time rate of change of the first moment of the vorticity field. By applying the Taylor hypothesis, *dx* = *U*_*c*_ *dt*, one can transform the spatial derivative in Eq (32) into a temporal one.

In the unsteady thin airfoil terminology the circulatory lift component is only a portion of the total lift that acting on the flapping wing. Since at the beginning of the flapping cycle the lift is unknown we refer to the estimated lift component as an increment in the circulatory lift that is generated from the beginning of the cycle, thus equal to Δ*L*_*c*_(*t*) and can be expressed as

$$\Delta L_{c}\left(t\right)=\rho U_{\infty }\int \zeta (t)\;dt.$$ (33)

In order to estimate the circulatory lift Δ*L*_*c*_(*t*) from Eq (33) one needs to acquire information regarding the vorticity flux *ζ*(*t*) in the near wake

$$\zeta \left(t\right)=\int U_{c}\omega_{z}\left(t\right)\;dy.$$ (34)

**Fig 6:**
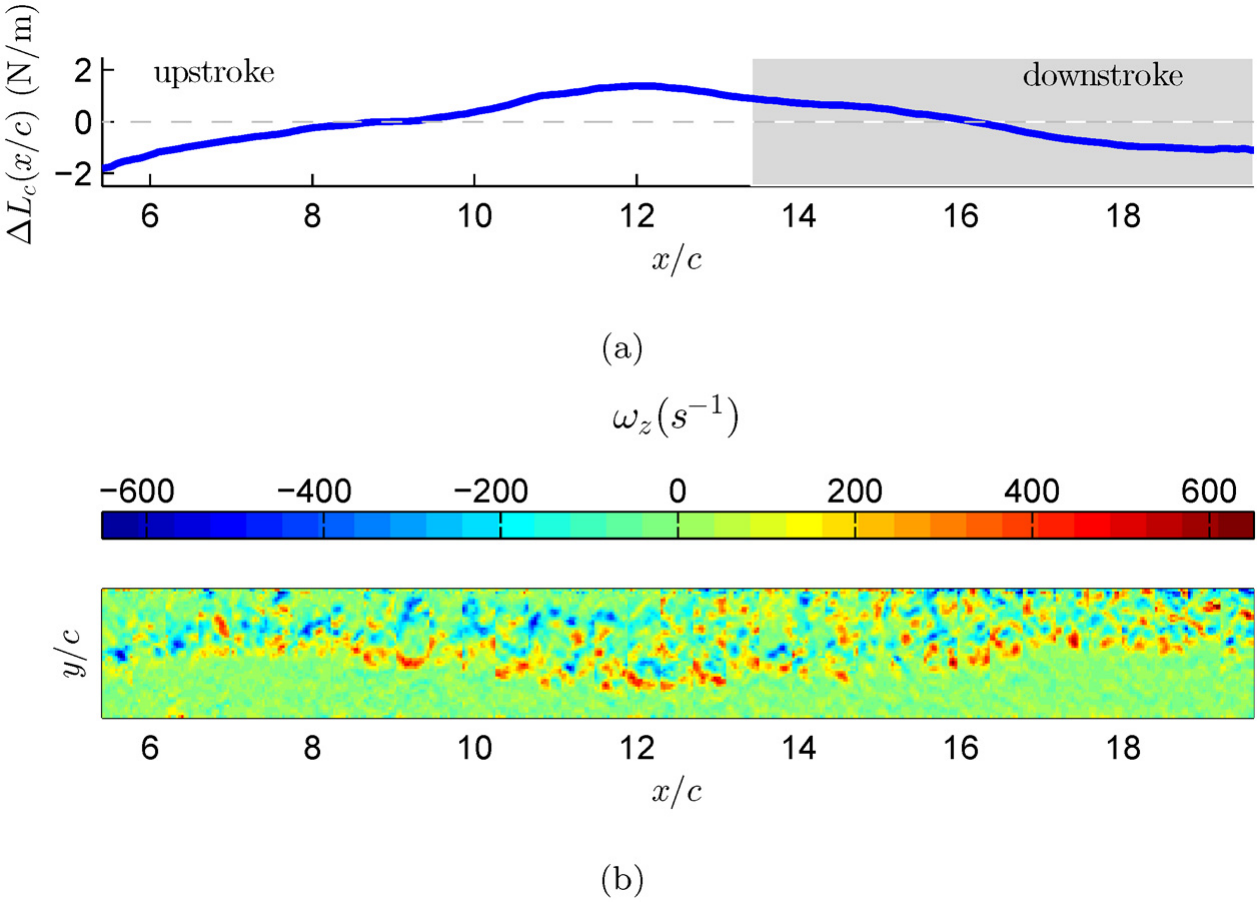
Estimation of circulatory lift component based on wingbeat number 1. (a) The circulatory lift was estimation based on Eq (33). The grey area indicates downstroke flapping phase. (b) Reconstruction of the starling’s wake vorticity as thought the bird flies from right to left.

**Fig 7:**
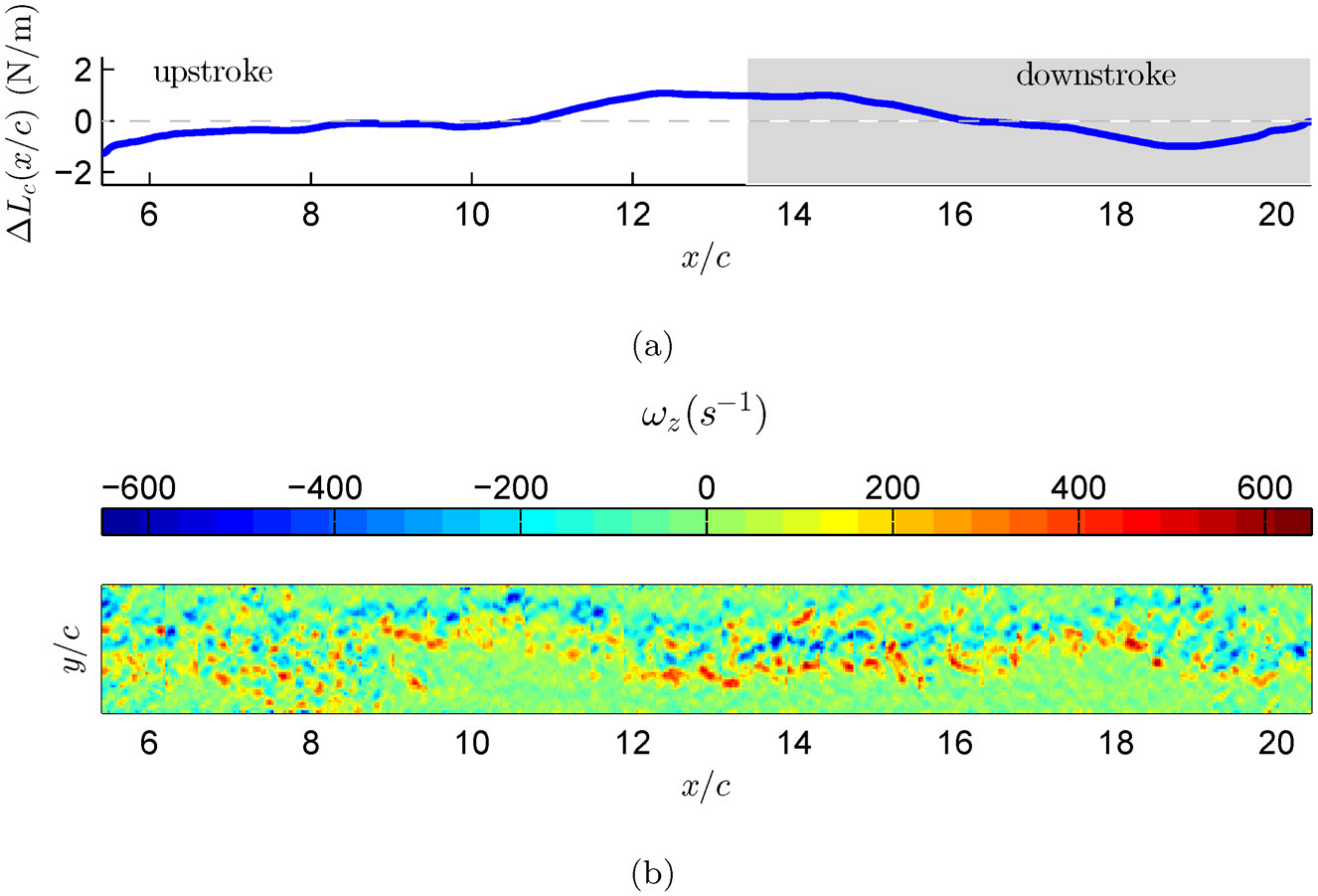
Estimation of circulatory lift component based on wingbeat number 2. (a) The circulatory lift was estimation based on Eq (33). The grey area indicates downstroke flapping phase. (b) Reconstruction of the starling’s wake vorticity as thought the bird flies from right to left.

**Fig 8:**
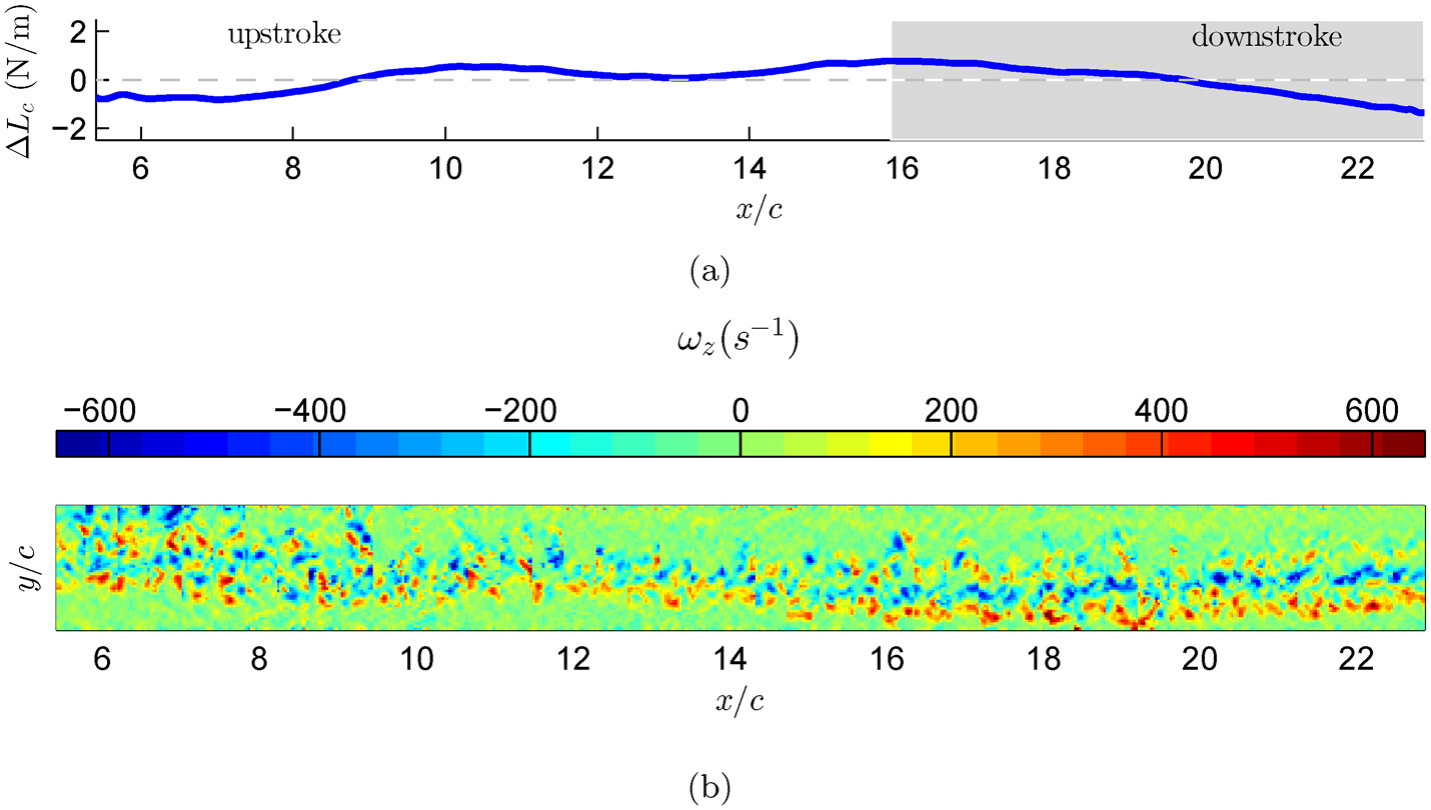
Estimation of circulatory lift component based on wingbeat number 3. (a) The circulatory lift was estimation based on Eq (33). The grey area indicates downstroke flapping phase. (b) Reconstruction of the starling’s wake vorticity as thought the bird flies from right to left.

The vorticity flux, defined by Eq (34), is estimated for each individual vector map as function of time. The calculated vorticity flux corresponds to the spanwise vorticity component and is integrated over a selected region in each vector map. The selected region covers the wake features that are observed in Figs 6(b), 7(b), and 8(b). Fig 6(a) demonstrates the changes in the circulatory lift component as it evolves over time, calculated based on Eq (33) over a single wingbeat cycle. This calculation was performed for three different wingbeat cycles. The curve represents the cumulative lift over one wingbeat cycle, starting from right to left as the bird is moving from right to left in respect to the coordinate system. Overall, the lift accumulates positively during the downstroke phase whilst negative accumulation is depicted during the upstroke phase. One can deduce that the circulatory lift has a positive net effect during the downstroke phase, which is in agreement with former work [42, 43]. During the upstroke phase, it appears that the circulatory lift is decreasing; this implies that during this phase, the bird is losing energy through the unsteady mechanism. Furthermore, the presence of cumulative negative circulatory lift during the upstroke phase marks the energy that the bird has to invest in order to bring the wing back to the downstroke phase to generate lift, again.

In the case of flapping flight of natural free-flyers, the wings’ motion is extremely complicated and it comprises from a complex flapping motion (changes in effective angle of attack), wing deformation (the bird stretches or bending its wing) and substantial three dimensional motion [44], resulting in a complex wake vorticity system [19]. As was shown earlier by the unsteady thin airfoil theory, the vorticity is shed into the wake continuously and affects the total circulation around a lifting surface [6, 7]. Therefore, as demonstrated in Figs 6, 7, and 8, the evolution of the lift over a single wingbeat cycle should be considered even for the case of power estimates where it is shown that while on average the lift should be equal to the bird’s weight, the time dependent variations of the lift from this value might provide a plausible argument to a more efficient flight.

Pennyquick [45] suggested to use the quasi-steady approach when estimating lift in bird flight. In his work, power was estimated based on kinematic analysis of flying birds and some assumptions related to drag and lift. The lift was assumed to be equal to weight, as any body that is aloft and in equilibrium. Following this approach, Tucker [46] revisited this argument and refined it to consider the flapping motion of the wings. Based on these works, Rayner [47] proposed a mathematical model for lift estimation from the wake of a flying bird in various flight modes. This model is well supported in the literature and it is conceptually accepted that unsteady mechanisms are of minor importance since variation of wing pitch and circulation are not producing thrust [48]. Our results, on the other hand, demonstrate that the unsteady mechanisms indeed play a significant role in the generation of lift. Whilst, thrust is not generated by lift, it is the energy that is required to keep the bird aloft that is impacted by the lift mechanism, e.g., with less power required to generate lift, more power can be directed towards propulsion.

The argument that during the steady phase of flight the bird’s weight must be balanced by an equal amount of lift [45] is obviously valid for an average lift generated during a wingbeat cycle. In the current study the bird’s weight was equal to 2 N/m. Following this argument, for steady flapping flight, the starling is required to generate an equal amount of lift force. The three cycles show similar trends, where the lift variation over each wingbeat cycle is about ±2 N/m. As mentioned above, these lift values are the time variation of the circulatory lift component. Thus, in order to obtain the actual total lift force produced by the starling, one needs to add these fluctuations to the bird’s weight that represents the averaged lift over a wingbeat cycle. By adding the lift fluctuations with the starling’s weight we find that during the flapping cycle the starling can produce up to 4 N/m of lift force, which is also equal to twice its body weight. These maximum lift values are generated mostly during the downstroke to upstroke transition phase, as depicted in Figs 6(b), 7(b), and 8(b), which is in good agreement with Kirchhefer *et al*. [19] who showed particular flow structures (termed: ‘double branch features’) occurring during the downstroke to upstroke transition. In addition, one can observe that the the minimum lift values are produced during the transition from upstroke to downstroke phase. The physical argument to this observation is that during the upstroke to downstroke transition the starling folds its wings, causing them to stop acting as lifting surfaces, and thus generates almost no lift. The aforesaid results imply that the usage of quasi-steady lift theory might be underestimating the lift that a bird is actually generates during flapping flight.

## Conclusions

The objective of this study was to evaluate the effect of bird generated flapping motion on the generation of unsteady lift. A freely flying European starling’s kinematics and near wake flow fields were acquired simultaneously at the AFAR facility. The bird’s kinematic motion was measured with a high speed imaging system, whilst the near wake flow field was acquired with long–duration time–resolved PIV.

To estimate the time dependent contribution to the lift we have applied unsteady thin airfoil theory, as developed by Theodorsen [6], and von-Kármán and Sears [7]. This theory addresses the various mechanisms that contribute to the time dependent lift components: the quasi-steady, added mass, and wake-induced vorticity. Using these terms, we have estimated the lift from the wingbeat kinematics and demonstrated that the contribution of each one of the terms to the total lift is not negligible.

An alternative approach to estimate the lift was based on the work of Wu [28] who derived the aerodynamic forces directly from the momentum equations. The aerodynamic forces were estimated from the wake flow field measurements, as acquired with PIV, and provided an estimation of the unsteady lift component. As discussed by Panda and Zaman [29] only the circulatory lift component can be estimated from the wake measurements. The effect of non-circulatory (or added mass) contribution could not be predicted from the wake measurements.

In this work, three wingbeat cycles were analysed. The near wake behind the birds wing was spatially reconstructed from the temporal velocity fields. The reconstructed wake field is spanning over 20 chord lengths downstream and depicts the flow features in the wake. The circulatory lift component over the wingbeat cycle follows the flow features as shown by the reconstructed wake. The evolution of the circulatory lift presents a negative contribution during the upstroke phase, whilst during the downstroke phase a positive contribution is observed. In addition, we observed that the downstroke phase is shorter, compared to the upstroke phase. This asymmetrical pattern is essential for the production of the high lift impulse that supports the bird’s weight. We conclude that the bird prefers to generate high lift values with a short downstroke phase than moderate lift values with a longer downstroke phase.

The two methods show that the unsteady component of the lift during flapping motion is not negligible. Both methods demonstrate that the time dependent components vary over the wingbeat cycle and contribute to the total lift as generated by the bird. Thus, we suggest that the time dependant lift component cannot be assumed negligible and should be considered when estimating lift (or power) of birds in flapping motion.

The assumptions of planar wake and the validity of the Kutta condition in the inviscid thin airfoil theory highly simplify the complex flow physics. It also excludes wake roll-up, convection of large separations over the wing section, boundary-layer separation, large laminar separation bubbles, leading-edge and trailing-edge vortices, three-dimensional effects and so forth. However, these effects are significant at low Reynolds numbers. Furthermore, the assumption of the Kutta condition in unsteady flapping flight might not be possible. Thus, lift estimated by the unsteady thin airfoil theory only partial explains the complex flow physics.
